# Enhancing Antioxidant and Anti-Inflammatory Activities of Garlic by Puffing

**DOI:** 10.3390/molecules30092022

**Published:** 2025-05-01

**Authors:** Hye-Jung Yang, Jae-Sung Shin, Seon-Min Oh, Ji-Eun Bae, Sang-Jin Ye, Hyun-Wook Choi, Moo-Yeol Baik

**Affiliations:** 1 Department of Food Science and Biotechnology, Institute of Life Science and Resources, Graduate School of Biotechnology, Kyung Hee University, Seoul 17104, Republic of Korea; applehyejung@khu.ac.kr (H.-J.Y.); drumlover@naver.com (J.-S.S.); seonmin@kfri.re.kr (S.-M.O.); wise123@khu.ac.kr (J.-E.B.); ysj7153@korea.kr (S.-J.Y.); 2 Healthcare Business Unit, Samyang Foods, Seoul 02737, Republic of Korea; 3 Food Processing Research Group, Korea Food Research Institute, Wanju-gun 55365, Republic of Korea; 4 Department of Food Science and Nutrition, College of Fisheries Science, Pukyong National University, Busan 48513, Republic of Korea; 5 Department of Central Area Crop Science, National Institute of Crop Science, Rural Development Administration, Suwon 16613, Republic of Korea; 6 Department of Food and Nutrition, Jeonju University, Jeonju 55069, Republic of Korea

**Keywords:** garlic, puffing, black garlic, antioxidant activity, anti-inflammatory activity

## Abstract

Garlic (*Allium sativum* L.) is well known for its numerous health benefits; however, its strong flavor and aroma may limit its consumption due to gastrointestinal discomfort. This study aimed to evaluate puffing as a novel garlic processing method and compare the properties of puffed garlic with those of raw and black garlic (BG). Puffing was applied at different pressures after adjusting moisture content, resulting in significant physicochemical changes, including increased browning and the development of a porous, crunchy texture. In contrast, BG exhibited a black coloration and a gelatinous texture. Puffing resulted in a marked improvement in extraction yield (except at 196 kPa) and an increase in Maillard reaction products (MRPs), which correlated with the intensity of browning. Although the total phenolic content (TPC) and total flavonoid content (TFC) increased three-fold and eight-fold, respectively, compared to raw garlic, the antioxidant activities determined by DPPH and ABTS radical scavenging activities increased by 22-fold and 61-fold, respectively, compared to raw garlic. All garlic samples demonstrated anti-inflammatory activity, with puffing pressure significantly influencing the suppression of IL-6 production. While BG is widely recognized for its enhanced health-promoting properties, puffed garlic exhibited comparable antioxidant and anti-inflammatory benefits in a shorter time frame and with a better retention of garlic’s original flavor. These findings highlight puffing as an efficient and promising alternative to traditional garlic processing, enhancing functionality while reducing sensory drawbacks.

## 1. Introduction

Garlic (*Allium sativum* L.) has been cultivated for centuries and is widely recognized for its numerous health benefits, which are attributed to its diverse bioactive compounds. Its efficacy spans various domains, including cardiovascular protection [1], anticancer properties [2,3], antibacterial effects [4], and antioxidant and anti-inflammatory activities [2,5]. Garlic’s health-promoting effects are largely due to its high content of organosulfur compounds [6]. However, despite its functional properties, garlic’s strong odor, primarily resulting from volatile organosulfur compounds such as allicin, diallyl disulfide (DADS), allyl methyl disulfide, and methyl mercaptan, is often perceived as undesirable [7,8]. To mitigate this issue, various processing methods, including heat treatment, aging, and fermentation, have been employed to modify garlic’s sensory attributes and improve its palatability [9]. These processing techniques induce compositional and structural changes, such as color development via the Maillard reaction [10], the enzymatic conversion of organosulfur compounds [11], and increased phenolic and flavonoid contents [12,13].

Among processed garlic products, black garlic (BG) has gained significant attention due to its softened texture, reduced pungency, and enhanced bioactivity. The transformation of raw garlic into black garlic occurs through a fermentation-like aging process under high temperature and humidity. During this process, allicin undergoes thermal degradation and is converted into stable sulfur-containing compounds, such as S-allyl-L-cysteine (SAC) and S-allylmercaptocysteine [11]. These modifications contribute to the superior antioxidant properties of black garlic [14]. However, the production of black garlic typically requires an extended processing period of over 20 days, with variations depending on temperature and humidity conditions.

Puffing is a high-temperature, high-pressure processing technique that rapidly evaporates moisture, inducing expansion and the formation of a porous structure, thereby modifying the texture of food products [15]. This process also promotes the Maillard reaction, leading to browning and the development of volatile compounds that enhance the overall flavor profile [16,17]. Previous studies have shown that puffing significantly enhanced the antioxidant activity of various plant-based materials, including turmeric, ginger, coffee, ginseng, and soybeans [18,19,20,21,22,23]. Additionally, puffing has been reported to improve anti-inflammatory activity [18] and increase nitric oxide scavenging capacity [23].

Despite extensive research on black garlic regarding its improved sensory properties and bioactivity, no studies have investigated the potential of puffing as a processing method for garlic. Given the lengthy processing time required for black garlic production, this study investigated puffing as a rapid alternative to enhance garlic’s functional properties. Specifically, the effects of puffing on garlic’s physicochemical characteristics, antioxidant activity, and anti-inflammatory potential were evaluated to expand its applicability in the food industry.

## 2. Results and Discussion

### 2.1. Morphology and Extraction Yield

**Figure 1:**
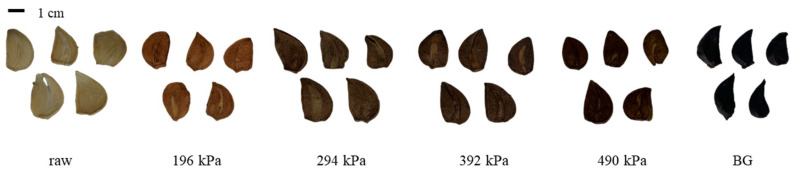
Morphology of raw, puffed, and black garlics.

The morphologies of raw garlic, puffed garlic, and BG are shown in Figure 1. Compared to raw garlic, puffed garlic exhibited a darkening effect as the pressure increased, possibly due to the Maillard reaction, a non-enzymatic browning reaction. During gun puffing, the inulin and fructans in garlic may break down into fructose and fructooligosaccharides. Concurrently, proteins may undergo denaturation and hydrolysis, resulting in the formation of amino acids. These sugars and amino acids contribute to the Maillard reaction, resulting in the darkening of puffed garlic with increasing pressure. Similar findings have been reported for red ginseng, where the degree of browning increased as the puffing pressure increased [24].

Although volume expansion is generally observed after puffing treatment, no volume expansion was observed in the case of garlic. Similarly, in the case of steam puffing, which is based on the pressure of moisture, the best puffing quality was observed for purple sweet potato when the moisture content ranged between 35% and 45% [25]. Additionally, the degree of gelatinization and crystallinity of starch significantly influences the outcomes of volume expansion. Common garlic has a moisture content of more than 60% and contains about 30% carbohydrates [26], with very little starch; over 70% of its carbohydrate content is inulin [27]. This may be a contributing factor to garlic’s limited expansion.

**Figure 2:**
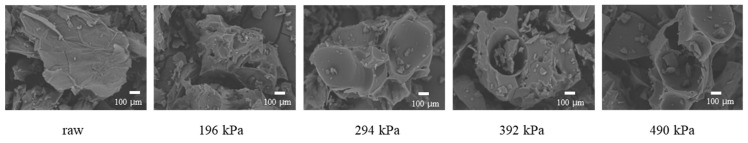
SEM images of raw and puffed garlics.

Figure 2 shows the internal structure of raw and puffed garlics. Initially, the surface area was large, but with the application of high heat and pressure, cracking occurred at 196 kPa, leading to the formation of a porous structure at 294 kPa. This indicates that while significant volume expansion did not occur, a porous structure was created due to rapid water evaporation.

The formation of a porous structure also affected the extraction yield (Table 1). Puffed garlic exhibited higher extraction yields than raw garlic, with the exception of puffed garlic at 196 kPa. Furthermore, the extraction yield increased with increasing pressure. This increase is possibly due to the increased contact surface area of the porous structure created by puffing, which facilitates the extraction of a greater number of active ingredients. Additionally, it is believed that puffing disrupts the cell wall substances that are strongly bound to the cell wall, allowing for the extraction of more compounds. Conversely, the extraction yield of BG was lower than that of raw garlic. It has been reported that when raw garlic is fermented at high temperatures for a long time, it contains high-molecular-weight melanoidins and Amadori products formed through the Maillard reaction, which are difficult to extract due to their complex structure and large molecular weight [28,29].

### 2.2. Maillard Reaction Products (MRPs)

The pressure inside the puffing chamber has a direct effect on the internal temperature. As pressure increases, the internal temperature also increases. Inulin, which constitutes the majority of carbohydrates in garlic, is hydrolyzed into monosaccharides such as fructose and glucose at high temperature and pressure. Simultaneously, proteins are also thermally degraded and increase the content of amino acids [30], which further accelerates the Maillard reaction. The levels of the Maillard reaction products (MRPs) of puffed garlic increased with increasing temperature and pressure (Table 1). The highest MRP content was observed in the dark-colored BG compared to both raw and puffed garlics. Maillard reaction intermediates, such as hydroxymethylfurfural (HMF) and melanogen, which are produced by the heat treatment of garlic, are known to increase with prolonged heating, contributing to the darkening of garlic’s color [10]. Although puffing involves high temperature and pressure, puffed garlic exhibited lower MRP levels compared to BG, likely due to the relatively short heating time.

### 2.3. Total Phenolic Content (TPC) and Total Flavonoid Content (TFC)

**Figure 3:**
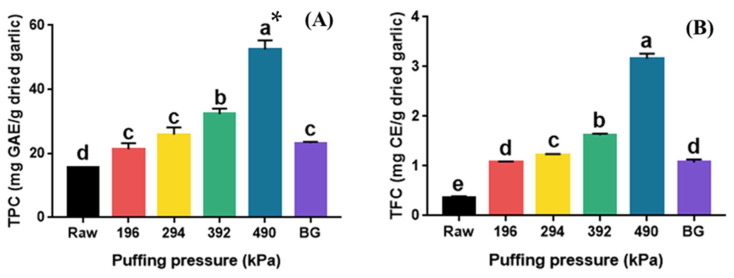
TPC (**A**) and TFC (**B**) of raw, puffed, and black garlics. * Different superscripts in same column indicate significant difference based on Tukey’s multiple range test (*p* < 0.05).

The total phenolic (TPC) and total flavonoid content (TFC) of raw, puffed, and black garlics are shown in Figure 3. The TPC of puffed garlic significantly increased in proportion to the puffing pressure (*p* < 0.05). Specifically, the TPC increased approximately three-fold, from 15.05 mg GAE/g dried garlic (raw garlic) to 52.69 mg GAE/g dried garlic (puffed garlic at 490 kPa). The puffing of natural products, such as turmeric, ginger, cultivated wild panax ginseng, black soybean, and ginseng berry, has been reported to increase the TPC compared to their raw materials [21,22,23]. The TPC of puffed ginger also proportionally increased with increasing puffing pressure and showed a two-fold increase at 980 kPa compared to the control [19]. On the other hand, the TPC of BG was 23.19 mg GAE/g dried garlic, which is 1.5 times higher than that of raw garlic. It has been reported that the TPC of BG at different thermal processing steps also increased from 105.73 mg GAE/kg to 982.14 mg GAE/kg [31].

The TFC of puffed garlic (1.08–3.16 mg CE/g dried garlic) also significantly increased with increasing puffing pressure (*p* < 0.05). TFC increased approximately nine-fold from 0.37 mg CE/g dried garlic (raw garlic) to 3.16 mg CE/g dried garlic (puffed garlic at 490 kPa). Similarly to the TPC of BG, the TFC of BG also increased to 1.08 mg CE/g dried garlic, which is about 2.9 times higher than that of raw garlic. The TFC of BG at various thermal processing steps also increased from 595.38 mg GAE/kg to 869.94 mg GAE/kg, which was about a 1.5 times increase [32]. Flavonoids, known as the secondary metabolites in plants, are disintegrated by heat from macromolecules into smaller molecules, resulting in an increase in TFC [19]. Moreover, the TFC of various fruit juices increased after high-pressure homogenization due to the destruction of cellular structures [19]. The increase in TFC in heat-processed garlic was reported to be due to the breakdown of large molecules containing polymeric flavonoids into smaller molecules [33]. It has also been reported that the puffing treatment of ginseng disrupted the cell structure, resulting in an increase in flavonoid content [34]. Consequently, the increase in the TFC of puffed garlic is likely a result of the heat and pressure applied during the puffing process, which destroyed the cell structure and facilitated the breakdown of various polymers containing flavonoids.

### 2.4. Antioxidant Activity

**Figure 4:**
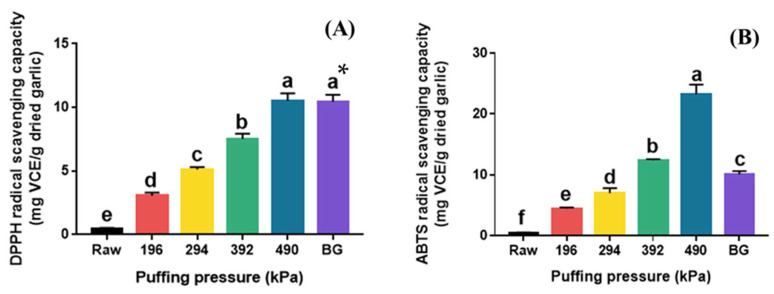
DPPH (**A**) and ABTS (**B**) radical scavenging capacities of raw garlic, puffed garlic, and black garlic (BG). * Different superscripts in same column indicate significant difference based on Tukey’s multiple range test (*p* < 0.05).

The DPPH and ABTS radical scavenging activities of raw, puffed, and black garlics are shown in Figure 4. Raw garlic showed very low DPPH radical scavenging activity (0.44 mg VCE/g dried garlic), and a significant increase was observed in puffed garlic with increasing pressure (Figure 4A). The highest DPPH radical scavenging activity was observed at 490 kPa (10.51 mg VCE/g dried garlic), which represents a 23-fold increase compared to that of raw garlic. Interestingly, there was no significant difference in DPPH radical scavenging activity between BG and puffed garlic at 490 kPa. The TPC and TFC of BG were significantly lower than those of puffed garlic at 490 kPa. These results suggest that DPPH radical scavenging activity may not be significantly affected by TPC or TFC in this case.

The ABTS radical scavenging activity of puffed garlic also increased with increasing puffing pressure (Figure 4B). Puffed garlic showed the highest ABTS radical scavenging activity at 490 kPa (23.26 mg VCE/g dried garlic), which is 61 times higher than that of raw garlic (0.38 mg VCE/g dried garlic). Although BG also had a much higher ABTS radical scavenging capacity (10.09 mg VCE/g dried garlic) than that of raw garlic, there was a notable difference between puffed garlic and BG, unlike the DPPH radical scavenging activity. This result indicates that DPPH and ABTS radical scavenging activities were controlled by different modes of action. DPPH radical scavenging activity is a commonly used method for evaluating the antioxidant activity of substances, particularly plant extracts, by measuring their ability to neutralize free radicals, as indicated by a color change from purple to yellow. Meanwhile, ABTS radical scavenging activity determines the color loss of the dark blue ABTS •+ cation and its change to colorless ABTS when antioxidants are added. On the other hand, 5-HMF, a major MRP, is known to significantly enhance DPPH radical scavenging activity [35]. Therefore, it is believed that the increase in MRPs in puffed garlic contributed to the enhanced DPPH radical scavenging activity.

### 2.5. Anti-Inflammatory Activity Test

**Figure 5:**
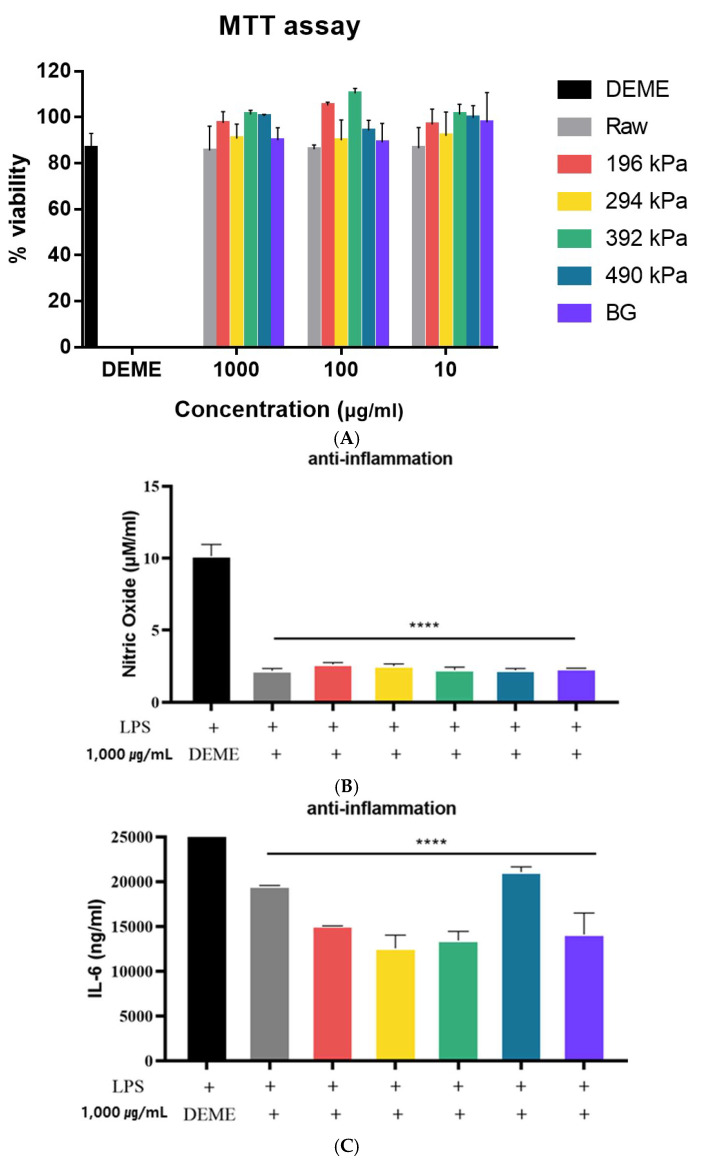
Anti-inflammatory activities of raw, puffed, and black garlics: (**A**) cytotoxicity, (**B**) NO production, and (**C**) IL-6 production. **** *p* < 0.0001 compared with LPS group

The viability of the RAW 264.7 cell line following treatment with raw, puffed, and black garlic extracts was evaluated using the MTT assay. As shown in Figure 5A, over 80% cell viability was observed after treatment with raw, puffed, and black garlic extracts, with no statistically significant differences compared to the DMEM-treated control cells (*p* > 0.05). This indicates that raw, puffed, and black garlics are non-toxic to cells at a concentration of 1000 μg/mL. Similarly, both raw garlic and black garlic extracts exhibited IC50 values greater than 1000 μg/mL, indicating very low cytotoxic activity [36]. Additionally, the survival rate was 100% when BG was administered at the highest concentration of 100 μg/mL [37]. Therefore, extracts at a concentration of 1000 µg/mL were used in the subsequent assessments of anti-inflammatory activity.

The effects of raw, puffed, and black garlic extracts on nitric oxide (NO) production in LPS-activated RAW264.7 cells are shown in Figure 5B. All samples significantly reduced LPS-induced NO production by 70% (*p* < 0.0001), indicating that puffing did not significantly influence anti-inflammatory activity. This result indicated a greatly beneficial role of raw, puffed, and black garlic extract treatments in modulating the production of the inflammatory mediator NO in macrophages. Meanwhile, no difference in NO production among the raw, puffed, and black garlic extracts may be attributed to the relatively high concentration of the extracts used. Typically, concentrations of 0.25–1 mg/mL are employed to assess anti-inflammatory activity, which is a lower dosage compared to the current study’s concentration of 1000 µg/mL [34].

The effects of raw, puffed, and black garlic extracts on the production of IL-6 inflammatory cytokines in LPS-induced RAW 264.7 cells are shown in Figure 5C. IL-6 production was reduced in all cells treated with raw, puffed, and black garlic extracts compared to those treated with LPS alone, indicating the anti-inflammatory properties of raw, puffed, and black garlics. Furthermore, puffed garlic at 294 kPa showed a further reduction in IL-6 production compared to cells treated solely with LPS. The results demonstrate that garlic extracts, irrespective of the puffing and fermentation processes, have an inhibitory effect on IL-6 production. Additionally, a specific puffing condition may enhance the anti-inflammatory properties of garlic. Overall, the anti-inflammatory activity of raw, puffed, and black garlics was observed in both NO and IL-6 production.

### 2.6. Quantification of S-Allylcysteine (SAC)

Table 2 shows the S-allylcysteine (SAC) content of fresh, puffed, and black garlics. As the heat and pressure increased, the SAC content decreased from raw garlic (57.86 μg/g) to puffed garlic at 490 kPa (4.65 μg/g) and BG (23.14 μg/g). From the previous results, the antioxidant activity of puffed garlic increased, and the content of total phenolic and flavonoid compounds also increased. However, anti-inflammatory activity did not correspondingly increase with increasing puffing pressure. This discrepancy arises because anti-inflammatory activity is regulated by complex biological pathways, including the modulation of inflammatory mediator expression, rather than merely the scavenging of oxides [38]. Certain organosulfur compounds, such as S-allylcysteine (SAC), play an important role in anti-inflammatory effects. Consequently, the structure of these compounds may have been modified or degraded during high-temperature and -pressure treatment, resulting in an insignificant enhancement in the anti-inflammatory activity of puffed garlic, despite the significant increase in TPC, TFC, and antioxidant activity compared to raw garlic.

## 3. Resources and Methods

### 3.1. Materials and Chemicals

Garlic harvested in 2022 was purchased from a local market in Uisung-gun, Republic of Korea. For this study, 1 M hydrochloric acid; 2,2-diphenyl-1-picrylhydrazyl (DPPH); 2,2′-azinobis(3-ethylbenzothiazoline-6-sulfonic acid) (ABTS); 2,2′-azobis(2-amidino-propane) dihydrochloride (AAPH); ascorbic acid; lipopolysaccharide (LPS); the Folin–Ciocalteu phenol reagent; gallic acid; and catechins were obtained from Sigma-Aldrich Co. (St. Louis, MO, USA). Phosphate-buffered saline (PBS) was acquired from Fisher Scientific (Waltham, MA, USA). Aluminum chloride and sodium nitrite were purchased from Junsei Chemical (Tokyo, Japan). L-ascorbic acid was purchased from Reagent Deoksan (Ansan, Republic of Korea). Dulbecco’s Modified Eagle’s Medium (DMEM) was obtained from Welgene (Gyeongsan, Republic of Korea). Enzyme-linked immunosorbent assay (ELISA) kits for IL-6 were purchased from BD-Bioscience (San Diego, CA, USA). Ethanol, methyl alcohol, and sodium hydroxide were purchased from Daejeong Chemical Metals (Siheung, Republic of Korea). All chemicals and reagents were of analytical grade and were used without further purification.

### 3.2. Preparation of Puffed Garlic and Black Garlic

The puffing treatment was conducted with modifications to the method described by Kim et al. [38]. A rotary expander (SHIN HAK, Seoul, Republic of Korea) was used for the puffing process. Sliced garlic was placed in a convection oven at 60 °C for 24 h to reduce the moisture content to 6.5%. The puffing process was initiated when the internal chamber pressure reached 196 kPa, 294 kPa, and 392 kPa, followed by a sudden pressure release upon opening the chamber door. For comparison, raw garlic was used in its dried and non-expanded state. Black garlic was prepared by washing the raw garlic, drying its surface, placing it in an electric rice cooker (Cuckoo Electronics Co., Yangsan, Republic of Korea), and then maintaining a temperature of 60–80 °C for 2 weeks [32].

### 3.3. Scanning Electron Microscopy (SEM)

The appearance of each sample was photographed using a camera (Apple Inc., Cupertino, CA, USA). The internal and microstructural characteristics of both raw and puffed garlics were examined using a scanning electron microscope (SEM, TM3000, Hitachi, Tokyo, Japan). Raw and puffed garlic samples were ground using a grinder (SFM-353NK, Shinil Industrial Co., Cheonan-si, Republic of Korea). Subsequently, all samples were placed on conductive carbon tape and coated with a gold–palladium coater (60:40) to enhance conductivity. Micrographs of each sample were obtained at an accelerating voltage of 15 kV.

### 3.4. Extraction Yield

Raw, puffed, and black garlics were extracted after fine grinding. Garlic (0.5 g) was mixed with 30 mL of 70% ethanol and extracted for 2 h at room temperature. The extracts were filtered using Whatman No. 2 filter paper, and the extraction yields were measured by drying sample aliquots (1 mL) at 105 °C.

The extraction yield was calculated using the following equation:

(1) $$extraction\;yield(\%)=\frac{\left(W_{2}-W_{1}\right)}{A}\times 100$$

where *A* is the weight of the garlics (g).

*W*_1_ is the initial weight of the plate (g).

*W*_2_ is the weight of the dish and the dried extracts (g).

### 3.5. Maillard Reaction Products (MRPs)

To determine the Maillard reaction products (MRPs), the extract was diluted to a concentration of 0.01 mg/mL using distilled water, and the absorbance was measured at 420 nm using a UV-Vis spectrophotometer (UV-12, LABENTECH Co., Incheon, Republic of Korea) [19].

### 3.6. Total Phenolic Content (TPC)

Folin and Ciocalteu’s phenol reagent (0.2 mL), distilled water (2.6 mL), and the extract (0.2 mL) were mixed. After 6 min, 2 mL of 7% Na_2_CO_3_ was added and allowed to react at 37 °C for 90 min. The absorbance was measured at 750 nm using a UV-Vis spectrophotometer(UV-12, LABENTECH CO., Incheon, Korea), with gallic acid serving as a standard material. The TPC results were expressed as mg gallic acid equivalents per gram of dried garlic [39].

### 3.7. Total Flavonoid Content (TFC)

Diluted extracts (0.5 mL), distilled water (3.2 mL), and 0.15 mL of 5% NaNO were mixed. After 5 min, 0.15 mL of 10% AlCl3 was added. After 1 min, 1 mL of 10% Na_2_CO_3_ was added. The absorbance was measured at 510 nm using a UV-Vis spectrophotometer, with catechin serving as the standard material. The TFC results were expressed as mg catechin equivalents per gram (mg CE/g) of dried garlic [19].

### 3.8. Antioxidant Activity

DPPH radical scavenging activity was assessed according to the method described by Brand-Williams et al. (1995), with minor modifications [40]. A DPPH solution (0.1 mM) was prepared in 80% methanol. The working solution was obtained by mixing 80% methanol to achieve an absorbance of 0.65 ± 0.02 units at 517 nm using a spectrophotometer. A sample of 0.05 mL was mixed with 2.95 mL of the 0.1 mM DPPH solution and kept in the dark for 30 min. After 30 min, the absorbance was measured at 517 nm using a UV-Vis spectrophotometer.

For the assessment of ABTS radical scavenging activity [41], a diluted extract (20 μL) was mixed with an ABTS •− radical solution (980 μL) and allowed to react for 10 min at 37 °C [42]. After the reaction, the absorbance was measured at 734 nm using a UV-Vis spectrophotometer. Vitamin C was used as a standard for both DPPH and ABTS radical scavenging activities. The results were expressed as vitamin C equivalents (VCE) per gram of dried garlic (mg/g).

### 3.9. Cell Culture and Processing

Cells were prepared at a density of 1 × 10^5^ cells/mL in Dulbecco’s Modified Eagle’s Medium (DMEM) supplemented with 10% fetal bovine serum (FBS) and 1% antibiotic–antimycotic solution (10,000 U/mL) [43]. Then, they were cultured in 24-well plates maintained at 37 °C in a humidified atmosphere of 95% air and 5% CO_2_ (model BB15; Thermo Scientific, Waltham, MA, USA). After 24 h, macrophages were stimulated with LPS at a concentration of 500 ng/mL for 12 h to induce an inflammatory state in RAW 264.7 cells. The culture medium was subsequently collected by centrifugation at 300× *g* for 5 min.

### 3.10. Cytotoxicity Assay

The cytotoxicity of garlic in RAW 264.7 cells was assessed by an MTT assay. Briefly, cells (2 × 10^5^ cells/mL) were seeded, exposed to garlic (10, 100, or 1000 μM) for 24 h, and then incubated with LPS (500 ng/mL) for an additional 24 h. After treatment with garlic, MTT solution (5 mg/mL) was added at a volume of 10 μL per well, and the cells were incubated for 3 h at 37 °C. Subsequently, the culture supernatant was removed, and MTT formazan was prepared in 100 μL of DMSO. The absorbance was then measured at 595 nm using a microplate reader (Bio-Rad, Hercules, CA, USA) to determine the cytotoxicity of garlic.

### 3.11. Measurement of Nitric Oxide

Nitrite concentration was measured using the Griess reagent system kit from Promega. Cells were exposed to lipopolysaccharides for 24 h subsequent to treatment with garlic extract in 96-well plates, each containing 2 × 10^4^ cells per well. Samples (50 μL) of the supernatant from the treated culture medium were mixed with 50 μL of 1% sulfanilamide in 5% phosphoric acid and incubated at room temperature for 10 min, shielded from light. After that, a 0.1% solution of N-1-naphthylethylenediamine dihydrochloride (50 μL) was added to the mixture. This solution was kept at room temperature for 10 min under light-shielding conditions. The absorbance was measured at a wavelength of 540 nm. Nitric oxide (NO) levels in each experimental sample were quantified using a sodium nitrite (NaNO_2_) standard curve ranging from 0 to 100 μM.

### 3.12. Measurement of IL-6 Production

The cellular secretion of the inflammatory cytokine IL-6 was assessed using ELISA kits (eBioscience, San Diego, CA, USA) according to the method described by Lee et al. [33]. Captured antibodies in a coating buffer were placed into a 96-well plate and stored at 4 °C overnight. The coating buffer was then removed, and the 96-well was washed 3 times with coating buffer. The 96-well plate was washed three times with a diluent after incubation with 200 µL assay buffer for 1 h. Standards or cell culture supernatants (100 µL) were added and incubated for 2 h. The 96-well plate was subsequently washed five times with the diluent and reacted with 100 µL of biotinylated detection antibody and avidin-conjugated horse radish peroxidase for 1 h. After the reaction, the 96-well plate was washed seven times with excess diluent, and 100 µL of substrate solution was added to each well and incubated for 30 min in the dark. After adding 50 µL of stop solution, the absorbance was measured at 450 nm using a spectrophotometer.

### 3.13. HPLC Analysis

The content of S-allyl-L-cysteine was determined using the method described in a previous study [44]. In summary, 100 mL of the supernatant was diluted with 900 mL of a methanol solution in water. After passing through a 0.45 μm membrane filter, the samples were analyzed using gradient elution on a C18 column (Prontosil 120-5-C18-SH 5.0 micrometer, measuring 150 mm × 4.6 mm) with a high-performance liquid chromatography (HPLC) system (Waters 2695 Alliance, Waters Inc., Milford, MA, USA). Solvent A consisted of a 20 mM sodium tetraborate solution, while solvent B contained 10 mM dansyl chloride. The injection volume was 10 μL, the flow rate was 1 mL/min, and the peak of the l-wave was identified using a Waters 996 photodiode array detector (Waters Corporation, Milford, MA, USA) at a wavelength of 250 nm.

### 3.14. Statistical Analysis

All experiments were repeated at least three times. Statistical analyses were performed using SAS version 9.4 for Windows (SAS Institute, Inc., Cary, NC, USA). Tukey’s test and a one-way ANOVA were employed to evaluate the significance of differences among the experimental mean values (*p* < 0.05).

## 4. Conclusions

The effects of puffing on antioxidant and anti-inflammatory activities, as well as the physicochemical properties of garlic, were investigated in comparison to raw garlic and black garlic (BG) to reduce the gastrointestinal discomfort caused by the potent flavor and intense scent of garlic. The morphology of puffed garlic exhibited an increase in browning with an increase in pressure. The internal porous structure of the material was altered, resulting in a crunchy texture in puffed garlic. In contrast, BG transitioned to a black hue and displayed a damp, supple, and pliable gelatinous consistency. A notable increase in extraction yield was observed across all puffed garlic samples except at 196 kPa compared to raw garlic, while BG exhibited a lower extraction yield. In correlation with their visual attributes, MRP levels increased with puffing as browning increased, while BG displayed a higher content of MRPs compared to both raw and puffed garlics. Total phenolic content TPC) and total flavonoid content (TFC) showed three-fold and eight-fold increases, respectively, compared to those of raw garlic. Antioxidant activities, as determined by DPPH and ABTS radical scavenging activities, increased by 22-fold and 61-fold, respectively, compared to raw garlic. The anti-inflammatory activity of raw, puffed, and black garlics was observed in both NO and IL-6 production. The inhibition of the NO production of garlic was not affected by puffing or fermentation, while puffing pressure significantly influenced the inhibition of IL-6 production in puffed garlic. Consequently, the puffing of garlic greatly enhanced its antioxidant and anti-inflammatory activities compared to raw garlic while reducing its distinct garlic flavor. BG is recognized worldwide as a healthier food option due to its increased antioxidant and anti-inflammatory properties, whereas puffed garlic retains its characteristic garlic taste, although diminished, while its antioxidant and anti-inflammatory activities are significantly enhanced. The production of BG requires more time and energy compared to the puffing of garlic, indicating that puffing could be a novel process for enhancing the health benefits of garlic.

## Figures and Tables

**Table 1:** Extraction yields and Maillard reaction products of raw, puffed, and black garlics.

Puffing Pressure (kPa)	Extraction Yield (%)	Maillard Reaction Products (Absorbance)
raw	42.21 ± 2.09 ^d^*	0.094 ± 0.008 ^f^
196	36.23 ± 0.64 ^e^	0.263 ± 0.018 ^e^
294	49.63 ± 0.24 ^c^	0.351 ± 0.015 ^d^
392	52.88 ± 0.63 ^b^	0.462 ± 0.005 ^c^
490	58.84 ± 1.06 ^a^	0.643 ± 0.017 ^b^
BG	21.01 ± 0.78 ^f^	0.674 ± 0.029 ^a^

* Different superscripts in same column indicate significant difference based on Tukey’s multiple range test (*p* < 0.05).

**Table 2:** S-allylcysteine concentration of raw, puffed, and black garlics analyzed using HPLC.

Puffing Pressure (kPa)	S-Allylcysteine (μg/g)
raw	57.86 ± 3.45 ^a^
196	30.86 ± 1.46 ^b^
294	23.98 ± 0.15 ^bc^
392	17.78 ± 2.02 ^c^
490	4.65 ± 0.18 ^d^
BG	23.14 ± 0.61 ^bc^

Different superscripts in same column indicate significant difference based on Tukey’s multiple range test (*p* < 0.05).

## Data Availability

The original contributions presented in this study are included in the article. Further inquiries can be directed to the corresponding author(s).
